# Consanguinity and its socio-biological parameters in Rahim Yar Khan District, Southern Punjab, Pakistan

**DOI:** 10.1186/s41043-016-0049-x

**Published:** 2016-05-20

**Authors:** Hafiza Fizzah Riaz, Shaheen Mannan, Sajid Malik

**Affiliations:** Department of Animal Sciences, Human Genetics Program, Faculty of Biological Sciences, Quaid-i-Azam University, Islamabad, 45320 Pakistan

**Keywords:** Consanguinity, Inbreeding coefficient, Fertility, Sex ratio, Child mortality, Child morbidity, Genetic epidemiology, Rahim Yar Khan, Southern Punjab, Pakistan

## Abstract

**Background:**

Rahim Yar Khan (RYK) District is a multi-ethnic assemblage of both ancient and migrated communities in Southern Punjab, Pakistan. There is a paucity of knowledge on the bio-demographic structure of this endogamous population.

**Methods:**

We have carried out a cross-sectional epidemiological study in RYK District and recruited 2174 random Muslim married females. Detailed account of marital union types, level of consanguinity, and subject’s fertility, was taken.

**Results:**

The analyses of these data revealed that consanguineous unions (CU) were 58.46 %, rendering an inbreeding coefficient (IC-F) = 0.0355. The CU were observed to be significantly higher in subjects originating from rural areas, speaking Saraiki language, illiterate or having a religious/Madarsa education only, and belonging to nuclear family type. The rate of consanguinity was also higher in subjects whose husbands were engaged in unskilled manual or skilled manual jobs, and had consanguinity in the parental generation. Multivariate logistic regression analyses revealed that variables like Saraiki language, illiteracy, reciprocal marriages, and parental consanguinity, were the significant predictors of CU in the subject. Among the first cousin unions (which constituted 52 % of all marriages), parallel-cousin and patrilineal unions were in the majority (54 and 57 %, respectively), and father’s brother’s daughter type had the highest representation (31 %). The analyses further demonstrated that fertility and mean live-births were significantly higher in women who had CU compared to the non-consanguineous (NCU) group (*p* < 0.006); and significantly higher number of sons per women were born to the mothers who had CU compared with the NCU sample (*p* = 0.0002). However, there were no differences in the CU and NCU samples with respect to pre- or post-natal mortalities and child morbidities.

**Conclusions:**

The scientific findings in RYK District are distinct from the observations in other Pakistani populations and clue to a unique nature of this population. This study presents a comprehensive account of consanguinity and IC-F in RYK District and would be helpful in getting an insight into the structure of this population.

## Background

The study of consanguinity is a subject of interest for both social scientists and human biologists. Understanding the pattern of consanguinity is not only helpful in getting an insight into the socio-biological structure of populations, but is also pertinent to the health and disease variables of the populations [1] Lately, the applications of autozygosity mapping in the identification of genes for rare recessive disorders has drawn attention towards the detailed understanding of consanguinity, its impact on genome homozygosity, and its implications in the isolated and inbred populations [2, 3].

Consanguineous unions (CU) are generally common in the developing world and especially in the Islamic countries. High rate of consanguinity has been observed to be associated with low socioeconomic status, illiteracy, and rural residence [4, 5]. The pattern of specific types of CU and their associated variables, however, differ in different populations across the globe. Further, the epidemiological studies have shown that there was a significant excess of congenital anomalies in the offspring of the consanguineous couples. The CU resulted in significantly increased incidences of abortion and stillbirth. Further, reproductive losses (i.e., neonatal, post-neonatal, infant, less than 5 years, and pre-reproductive mortalities) have been observed to be remarkably higher in the consanguineous communities compared to the non-consanguineous counterparts [1, 3, 6].

With few exceptions, CU are widely practiced in Pakistan. Studies have shown that over the time, the popularity of such marriages is not declining in many sub-populations of the country [4]. There are several studies reporting the incidence of consanguineous marriages in various regions of Pakistan like northern Punjab [7, 8], Balochistan [9], southern Khyber Pakhtoonkhwa [10], and Kashmir [11]. The overall prevalence of consanguineous marriages ranged from 31.1 to 62 %. There is no study, however, available for Southern Punjab, which is a less developed region of the country. Hence, the main aim of the present study was to establish consanguinity and its socio-demographic variables in a representative population of Southern Punjab, i.e., Rahim Yar Khan (RYK) District. This study further investigated the differences in consanguineous and non-consanguineous samples with respect to parameters like subject’s fertility, live births, prenatal/postnatal mortality, and child morbidity.

## Methods

### Study population

RYK District lies at the southern extremes of Punjab province and borders with the Sindh province. It has an area of 11,880 km^2^ with an estimated population of 4.7 million [12, 13]. The District comprises four administrative divisions called *tehsils*, i.e., Sadiqabad, Rahim Yar Khan, Khanpur, and Liaquatpur. The major caste systems are Arain, Jutt, Rajput, and Gujjar, which migrated here during and after the creation of Pakistan in 1947. The old settlers of the region are the Joya, Wattoo, Daudpota, Balouch, Syed, and Pathan [12, 13]. The population of the district is predominantly Muslim (96.7 %), and the largest minority is Hindu (1.8 %). *Siraiki* is the major language (62.6 %), followed by *Punjabi* (27.3 %), and *Urdu* (2.9 %). The District has mainly an agro-based economy, and a majority of the population resides in rural areas. The literacy rate is 33.1 %, which is the lowest in Punjab province [12]. There are relatively better facilities of health care in RYK, but the impact of preventive care services like “maternal and child health services” and “immunization programs” are severely marred due to illiteracy, lack of awareness, and poor socio-economic conditions [12, 13].

### Subject recruitment

A random sample was collected through a descriptive epidemiological study carried out during 2010–2011. There were a total of 35 different sampling sites encompassing the major towns/villages across the four tehsils of RYK District. Subjects were approached at their places of residences/work or by visiting public places like community centers and hospitals. Usually, a local resource person and lady-health-visitor accompanied the survey team. Only the married females who were permanent resident of the District and consented to provide complete information were included. All the data were acquired through face-to-face contact with the respondent. A formal consent was obtained from each respondent or her husband/in-laws prior to questionnaire filling. General queries from the respondents about the survey and the outcomes of the present study were adequately addressed before data collection. A structured questionnaire was developed to acquire the data. Only a single woman was recruited from a particular household. There were an estimated >416,000 housing units in the district [12, 13]. Hence, the proportion of the sampled households in RYK population was at least 0.005. Information was gathered on socio-demographic variables, marital types, and fertility variables. The Hindu community of the District exclusively practices non-consanguineous unions; hence, the data of Hindu subjects were not included in the analyses.

### Definitions

All the marital union types were coalesced into two broad categories of consanguineous unions (CU) and non-consanguineous unions (NCU). The CU comprised marriages between double first cousins (DFC), first cousins (FC), first cousin once removed (FCOR), and second cousins (SC); while the NCU were marriages between second cousin once removed (SCOR), distantly related/*biradari* (DR), and non-related (NR) [1, 10].

The geographic origin of the subject was identified as rural or urban, which was based on the union council records. Data were also collected with respect to the “marriage arrangements”, i.e., arranged, reciprocal, and self-arranged/arranged-love marriages. “Arranged marriages” were those in which the parents/elders of the subject played a key role in identifying marriage partner; “reciprocal marriages” involved two exchange marriages in two families (called *watta-satta* in local language); and “self-arranged/arranged-love marriages” were those in which the subject herself identified the marriage partner and marriage was subsequently contracted with the consent of both families. “Family/household” types were defined as either “nuclear”, “grandparent-and-one-couple” or “extended”. Nuclear families comprised one couple and their children living in the same house. Grandparent-and-one-couple were nuclear families with grandparents living together. Extended families comprised two or more couples living in three or more overlapping generations in the same household. Self-explained professions of the husband were resolved according to the standard occupational categories established in the Pakistan Demographic and Health Survey [14]. Accordingly, the literacy was evaluated as formal schooling and in number of years of attending the school.

Data were analyzed through MS-Excel and GraphPad Prism (ver.5). Inbreeding coefficient-F (IC-F) was estimated from the weighted proportion of individual CU types [1, 10]. The *χ*^2^ test was employed for the comparison between categorical variables and 95 % confidence intervals were calculated for total frequencies. The *T* test and ANOVA were used to compare continuous variable means, because all had a normal distributions and comparable variances. For multivariate analysis, a stepwise likelihood ratio logistic regression was performed. Consanguinity was taken as dependent variables and the studied socio-demographic factors as independent variables. For bivariate regression analysis (odd ratios (OR)), the category with lowest rate of CU was taken as reference in each socio-demographic variable.

## Results

### Sample characteristics

A total of 2662 subjects were randomly approached during the fieldwork. However, 2174 individuals (82 %) gave their consent for the participation in this study. The recruited subjects ranged from 15 to 90 years in age (mean ± SD 35.2 ± 13.5). Among the four *tehsils*, 941 subjects belonged to Sadiqabad and there were 57 individuals from Liaquatpur (Table 1).

**Table 1:** Distribution of major types of marital unions in four tehsils of RYK District

	Consanguineous unions: no.(%)				Non-consanguineous unions: no.(%)			
Tehsils	Double first cousin	First cousin	First cousin once removed	Second cousin	Second cousin once removed	Distantly related/*Biradari*	Non-related	All marriages
Sadiqabad	11 (1.2)	477 (50.7)	23 (2.4)	16 (1.7)	5 (0.5)	345 (36.7)	64 (6.8)	941
Rahim Yar Khan	7 (1.2)	343 (57.4)	24 (4.0)	3 (0.5)	2 (0.3)	214 (35.8)	5 (0.8)	598
Khanpur	7 (1.2)	287 (49.7)	28 (4.8)	6 (1.0)	1 (0.2)	247 (42.7)	2 (0.4)	578
Liaquatpur	2 (3.5)	22 (38.6)	15 (26.3)	0	0	18 (31.6)	0	57
Total	27 (1.2)	1129 (51.9)	90 (4.1)	25 (1.2)	8 (0.4)	824 (37.9)	71 (3.3)	2174

### Consanguinity and inbreeding coefficient

In the total marriages, CU were observed to be 58.5 % (*n* = 1,271) while NCU were 41.5 % (*n* = 903) (Table 1). FC unions were the most prevalent type among the CU as well as in the total marriages (51.9 %; *n* = 1129). In the sample of subjects with NCU, the most common marriage type was DR (37.9 %; *n* = 824). The overall IC-F was established to be 0.0355 (Table 2).

**Table 2:** Distribution of consanguineous unions, total marriages, and IC-F across geographic location, mother tongue, and caste systems

Variable	Consanguineous unions		Total marriages		Bivariate logistic regression OR	IC-F
	No	%	No	%		
Tehsils*						
Sadiqabad	527	56.0	941	43.3	1.00	0.0342
Rahim Yar Khan	377	63.0	598	27.5	1.34*	0.0386
Khanpur	328	56.8	578	26.6	1.03	0.0342
Liaquatpur	39	68.4	57	2.6	1.70	0.0367
Total	1271	58.5	2174	100.0		0.0355
Rural/urban origin*						
Rural	844	60.7	1390	63.9	1.29*	0.0370
Urban	427	54.5	784	36.1	1.00	0.0327
Mother tongue*						
Punjabi	499	45.2	1105	50.8	1.00	0.0278
Saraiki	706	75.4	937	43.1	3.71*	0.0454
Urdu	23	46.0	50	2.3	0.99	0.0275
Others	43	52.4	82	3.8	1.66	0.0309
Caste system** (*n* = 1501)*						
Arain^a^	239	39.0	613	28.2	1.00	0.0239
Malik^b^	67	77.9	86	4.0	5.52*	0.0458
Arain^b^	54	80.6	67	3.1	6.50*	0.0483
Balouch^b^	50	58.1	86	4.0	2.17*	0.0338
Bhatti^b^	37	74.0	50	2.3	4.45*	0.0441
Jut^a^	49	45.0	109	5.0	1.28	0.0288
Khokhar^b^	41	91.1	45	2.1	16.04*	0.0535
Larr^b^	37	84.1	44	2.0	8.27*	0.0526
Malik^a^	28	58.3	48	2.2	2.19*	0.0332
Mughal^a^	22	51.2	43	2.0	1.64	0.0334
Rajput^a^	72	53.7	134	6.2	1.82*	0.0332
Sheikh^b^	37	80.4	46	2.1	6.43*	0.0530

^a^Predominantly *Punjabi* speaking

^b^Predominantly *Saraiki* speaking

^*Pushto* speaking

*Distribution was statistically significant; **caste systems with sample size ≥42 are shown

**Fig. 1:**
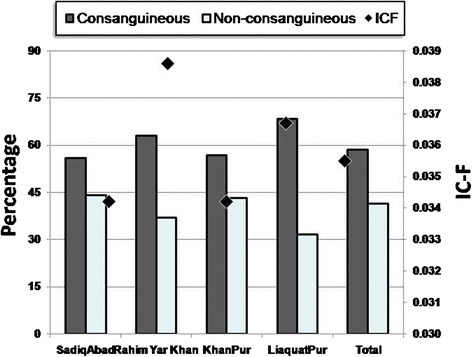
Bar graph depicting CU and NCU (*at the left Y-axis*) and IC-F (*in black diamonds*; *at the right Y-axis*) in four tehsils of Rahim Yar Khan District

Among the four *tehsils*, the proportion of CU was highest in the Liaquatpur *tehsil* (68.42 %). However, IC-F was highest in the RYK *tehsil* (0.0386) which was due to high proportion of FC unions; on the other hand, IC-F was observed to be 0.0367 in Liaquatpur (Table 2; Fig. 1). Furthermore, the differences in the distributions of CU and NCU among the *tehsils* were statistically significant.

### Consanguinity in socio-demographic variables

The CU were observed to be common in subjects belonging to rural areas compared to the urban counterparts (60.7 vs. 54.5 %, respectively), and their differences were statistically significant. With respect to the mother tongue of the respondent, the ratio of CU was most conspicuous in *Saraiki* speaking individuals (75.4 %; IC-F = 0.0454). Among the *Punjabi* and *Urdu* speaking individuals, CU were observed to be 45.2 and 46.0 %, respectively (Table 2).

There was considerable ethnic diversity in the sample and at least 150 minor caste systems were observed which were resolved into major caste systems. Among the prominent caste systems (*n* > 42), there was substantial heterogeneity in the rate of consanguinity. The frequency of CU was as high as >80 % in *Saraiki* speaking Khokhar, Arain, Larr, and Sheikh castes, and as low as >54 % in *Punjabi* speaking Arain, Jut, Mughal, and Rajput castes (*p* < 0.0001) (Table 2). Bivariate logistic regression analyses revealed that in most of the caste systems, the rate of CU was significantly higher than the reference group.

With respect to literacy, CU were more prevalent in the illiterate group (and in subjects having religious education only) compared to the literate sample (*p* < 0.0001). Among the literate subjects, a declining trend in the rate of CU and IC-F was evident with increasing literacy levels (Chi-square for trend; *p* = 0.1211) (Table 3). Further, the subjects were divided into three categories with respect to their ages. The CU were common in participants in the older age group. Further, there was a declining trend in the rate of consanguinity and IC-F with decreasing age ranges of the subjects (Chi-square for trend; *p* = 0.0124) (Table 3).

**Table 3:** Distribution of consanguineous unions, total marriages, and IC-F in various socio-demographic variables of subjects

Variable	Consanguineous unions		Total marriages		Bivariate logistic regression OR	IC-F
	No.	%	No.	%		
Education*						
Illiterate	564	65.6	860	39.6	2.33*	0.0396
Religious education/*Madarsa*	399	63.4	629	28.9	2.12*	0.0383
Literate (all)	308	45.0	685	31.5	1.00	0.0278
Literacy level (years of schooling)						
Up to 8 years	146	47.4	308	14.2	0.67	0.0301
9–12 years	128	44.6	287	13.2	0.89	0.0265
>12 years	34	37.8	90	4.1	1.00	0.0236
Subjects’ age group (years)						
Up to 25	342	54.7	625	28.8	1.00	0.0328
26–50	781	59.4	1315	60.5	0.75*	0.0363
>50	148	63.3	234	10.8	0.83	0.0379
Marriage arrangement (*n* = 2117)*						
Arranged marriage	736	50.2	1466	69.2	1.00	0.0306
Self-arranged	27	79.4	34	1.6	3.83*	0.0455
Reciprocal (*Watta-Satta*)	470	76.2	617	29.2	3.17*	0.0466
Family/household type (*n* = 2108)						
Nuclear	695	60.2	1154	54.7	1.12	0.0367
Grandparent-and-one-couple	292	57.5	508	24.1	1.00	0.0347
Extended family	244	54.7	446	21.2	0.89	0.0337
Occupation of husband*						
Unskilled manual	423	67.1	632	29.1	1.94*	0.0406
Agriculture/farming	288	59.9	481	22.1	1.45*	0.0362
Office job/services	239	51.7	462	21.3	1.04	0.0313
Business/sales	217	50.7	428	19.7	1.00	0.0315
Skilled manual	68	63.0	108	5.0	1.79*	0.0378
Unemployed	12	70.6	17	0.8	–	0.0368
Late/deceased	24	52.2	46	2.1	–	0.0313
Parental marriage type (*n* = 1998)*						
Consanguineous	725	74.7	970	48.5	4.05*	0.0453
Non-consanguineous	434	42.2	1028	51.5	1.00	0.0256

*Distribution was statistically significant

Regarding the marriage arrangements, the CU were higher in subjects having self-arranged and reciprocal marriages (Table 3). In the present sample, the most common household type was observed to be nuclear family. The rate of CU was observed to be higher in the subjects belonging to the nuclear family type. The rate of CU and IC-F was declining in grandparent-and-one-couple and extended family types.

With respect to husbands’ profession, the rate of CU was observed to be highest in subjects whose husbands were either engaged in ‘unskilled manual’ or ‘skilled manual’ jobs (IC-F = 0.0406 and 0.0378, respectively). The CU were also higher in subjects involved in ‘agriculture/farming’ (IC-F = 0.0362). On the other hand, consanguinity was lowest in husbands working in offices/services or engaged in businesses/sales (IC-F = 0.0313 and 0.0315, respectively).

### Effect of parental marriage type on subject’s marriage type

The data on parental marriage type was available for 1998 subjects. Among the parental generation, CU were observed to be 48.5 % (*n* = 970), and there were 51.5 % (*n* = 1028) NCU (Table 3). Inbreeding coefficient-F in the parental generation was 0.0351. The CU in parental generation led to a significantly higher rate of CU among the subjects; likewise NCU in the parental generation led to a significantly higher ratio of NCU among the subjects (*p* < 0.0001) (Table 3).

Bivariate logistic regression (odd ratios (OR)) iterated most of the results obtained through the contingency tests (Tables 2 and 3). The factors significantly associated with consanguinity were retained in the model of multivariate analysis (without caste systems): *Saraiki* language (OR = 2.01), illiteracy (OR = 1.31), religious education only (OR = 1.42), reciprocal marriage (OR = 1.82), and parental consanguinity (OR = 3.06) (Table 4). Interestingly, the variables like *tehsil*, rural/urban origin, age category, family type, and occupation of husband, became insignificant in the multivariate analyses. In another model (with caste systems), the significant variables were Khokhar caste (OR = 3.20), *Saraiki* language (OR = 1.93), illiteracy (OR = 1.41), reciprocal marriage (OR = 1.64), and parental consanguinity (OR = 2.87) (Table 4).

**Table 4:** Multivariate analysis of socio-demographic variables associated with CU

Socio-demographic variable	OR	Std.Err.	*P*	95 % CI
Model 1 (without caste system)				
Mother tongue				
Saraiki	2.01	0.26	<0.0001	1.55–2.60
Literacy				
Religious education	1.31	0.18	0.040	1.01–1.71
Illiterate	1.42	0.19	0.008	1.10–1.83
Marriage arrangement				
Reciprocal marriage	1.82	0.25	<0.0001	1.39–2.37
Parental marriage type				
Consanguineous union	3.06	0.32	<0.0001	2.50–3.75
Model 2 (with Caste-system)				
Caste-system				
Khokhar	5.41	3.20	0.004	1.70–17.25
Mother tongue				
Saraiki	1.93	0.61	0.038	1.04–3.59
Literacy				
Illiterate	1.41	0.22	0.024	1.05–1.91
Marriage arrangement				
Reciprocal marriage	1.64	0.29	0.006	1.15–2.33
Parental marriage type				
Consanguineous union	2.87	0.35	<0.0001	2.25–3.65

### Pattern of first cousin unions

Data on specific type of FC union was available for 1112 subjects (of the total 1129 marriages). Collectively, parallel-cousin unions were in the majority (*n* = 600; 54 %), as compared with the cross-cousin types (*n* = 512; 46 %) (Table 5). Further, patrilineal marriages were more common (*n* = 635; 57 %), compared with the matrilineal marriages (*n* = 477; 43 %).

**Table 5:** Types and distribution of first cousin unions in RYK population

Variable	First cousin marriage type				*n*	% in total marriages
	FBD	FSD	MBD	MSD		
*Tehsil*						
Sadiqabad	166	120	95	93	474	50.4
Rahim Yar Khan	107	90	64	81	342	57.2
Khanpur	76	72	68	71	287	49.7
Liaquatpur	1	3	0	5	9	15.8
Total	350	285	227	250	1112	51.1
Rural/urban origin						
Rural	250	189	146	154	739	53.2
Urban	100	96	81	96	373	47.6
Mother tongue*						
Punjabi	122	118	104	110	454	41.1
Saraiki	212	152	106	129	599	63.9
Education						
Illiterate	157	125	86	106	474	57.3
Religious education/*Madarsa*	112	81	76	77	346	54.8
Literate	82	81	65	68	296	41.3

*Distribution was statistically significant

Among the four FC union types, father’s brother’s daughter (FBD) marriages were the most prevalent (*n* = 350; 31 %), whereas mother’s brother’s daughter (MBD) marriages were the least common (*n* = 227; 20 %). The detailed distribution of FC unions by key demographic variables is presented in Table 5.

### Fertility and live births

**Fig. 2:**
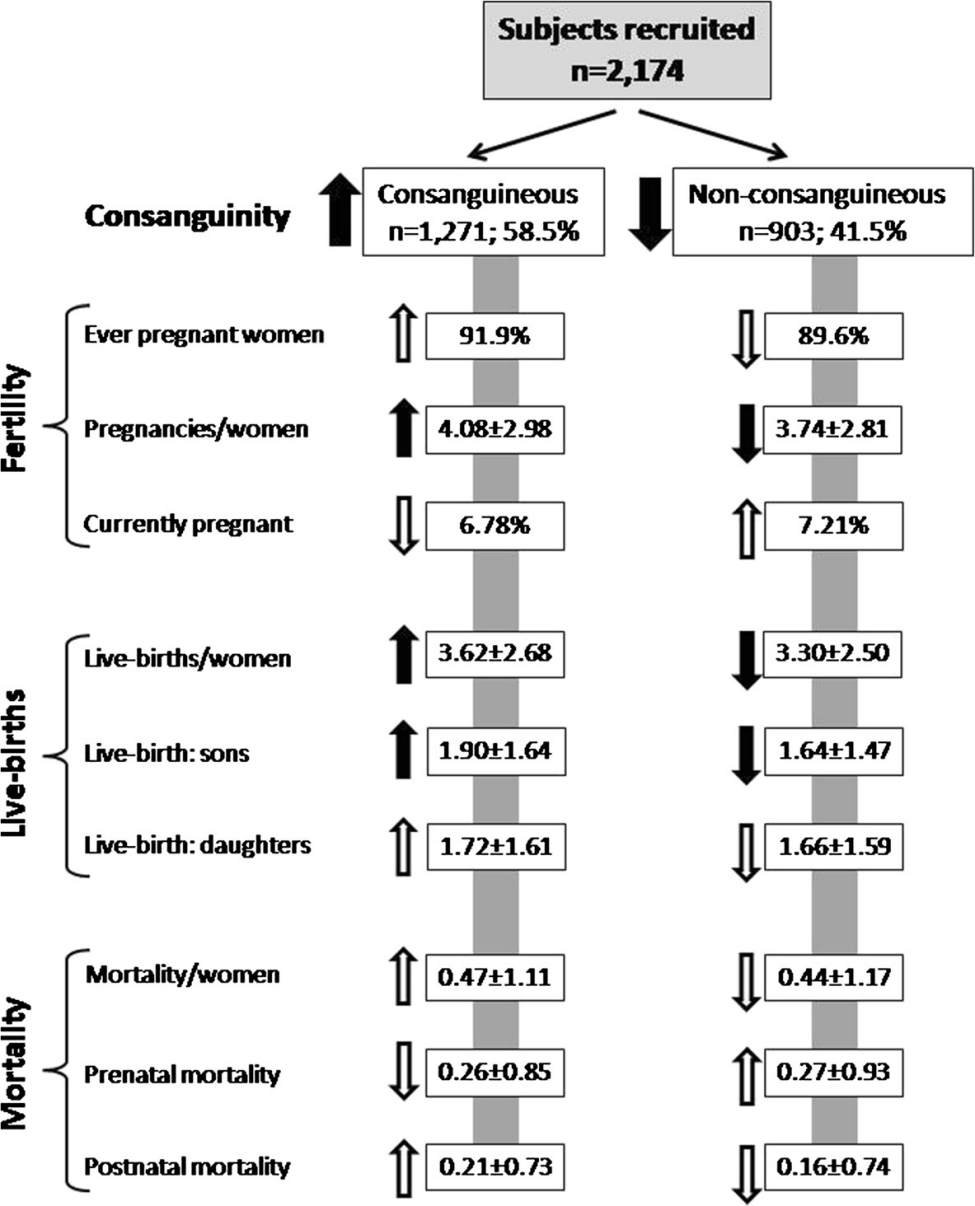
Flow chart showing the analyses scheme and the differentials in various parameters between consanguineous and non-consanguineous samples. *Arrows in upward direction* depict higher estimates in the respective sample and *downward* the lower estimates. *Black arrows* show that the differences in the distribution between the consanguineous and non-consanguineous samples were significant, while *white arrows* demonstrate non-significant distribution

There were a total of 1958 (90.90 %) ever pregnant women in the sample. The mean pregnancies per women were calculated to be 3.94 ± 2.91 (Table 4). The subjects having CU has a significantly higher fertility than the subjects having NCU (*p* = 0.007). The ever pregnant women delivered a total of 7503 live births, and the mean live births per women were calculated to be 3.48 ± 2.61. The number of live births per women were significantly higher in women who had CU compared with the subjects having NCU, and their differences were statistically highly significant (*p* = 0.0064). Further, significantly higher number of sons per women were born to the mothers who had CU compared with the NCU sample (*p* = 0.0002); however, there were no differences in the number of live-born daughters between the mothers with CU and the mothers with NCU (Table 6; Fig. 2).

**Table 6:** Subject’s fertility and live births in consanguineous and non-consanguineous unions

Parameter	Consanguineous union	Non-consanguineous union	Total	*p* value
Average age of subjects	34.99 ± 13.36	35.16 ± 13.31	35.06 ± 13.34	*t* 0.670
Fertility				
Ever pregnant women (no.)	1,138	820	1,958	
Ever pregnant women (%)	91.9	89.6	90.9	
Total pregnancies (no.)	5058	3423	8481	
Fertility: pregnancy/women (mean ± SD)	4.08 ± 2.98	3.74 ± 2.81	3.94 ± 2.91	*t*: 0.007*
Currently pregnant (no.)	84	66	150	
Currently pregnant (%)	6.78	7.21	6.96	
Live births				
Total live births (no.)	4479	3024	7503	
Live births/women (mean ± SD)	3.62 ± 2.68	3.30 ± 2.50	3.48 ± 2.61	*t* 0.006*
Live birth: sons (no.)	2350	1505	3855	
Live birth: sons (mean ± SD)	1.90 ± 1.64	1.64 ± 1.47	1.79 ± 1.58	*t* 0.0002**
Live birth: daughters (no.)	2129	1519	3648	
Live birth: daughters (mean ± SD)	1.72 ± 1.61	1.66 ± 1.59	1.69 ± 1.60	*t* 0.406

*Differences were highly significant; **differences were more highly significant (the statistical findings/significance did not differ when analyses were repeated through Mann-Whitney test and unpaired *t* test with Welch’s correction were performed)

### Prenatal mortality, postnatal mortality, and child morbidity

Among the ever pregnant women, there were a total of 978 mortalities/pregnancy losses. There were no differences in the average mortalities (i.e., prenatal, postnatal—within first year, and total) between the mothers with CU and with NCU. Similarly, the distribution of live-born child with certain type of congenital anomaly was statistically not significant between the mothers having CU and NCU (Table 7; Fig. 2).

**Table 7:** Prenatal and postnatal mortality and child morbidity in the consanguineous and non-consanguineous unions

Parameter	Consanguineous union	Non-consanguineous union	Total	*p* value
Mortalities				
Data available on mothers (no.)	1239	915	2,154	
Total mortalities (no.)	579	399	978	
Mortality/women (mean ± SD)	0.47 ± 1.11	0.44 ± 1.17	0.45 ± 1.14	*t* 0.528
Prenatal mortality (no.)	316	249	565	
Prenatal mortality (mean ± SD)	0.26 ± 0.85	0.27 ± 0.93	0.26 ± 0.88	*t* 0.658
Postnatal mortality (no.)	263	150	413	
Postnatal mortality (mean ± SD)	0.21 ± 0.73	0.16 ± 0.74	0.19 ± 0.73	*t* 0.169
Child morbidity (no.)				
Mortality in sons	43	36	79	*χ* ^2^ 0.295; OR 0.699 (CI 0.357–1.37)
Mortality in daughters	41	24	65	
Total mortality	84	60	144	

## Discussion

We report consanguinity and its associated variables in the population of RYK which is a remote District in Southern Punjab, Pakistan. The analyses revealed that among the four *tehsils* of the District, the rate of CU was highest in the Liaquatpur *tehsil* (68.42 %). However, the estimate of IC-F was most conspicuous in the RYK *tehsil* (0.0386), which had relatively low estimate of CU than Liaquatpur (i.e., 63 %). This discrepancy between the CU and IC-F could be explained by the observation that FC marriages were the most common type in the sample obtained from the RYK *tehsil* (57.4 %), whereas in the subjects from the Liaquatpur *tehsil,* there was a very high proportion of FCOR marriages and low representation of FC unions (Table 1). Furthermore, significantly higher rate of consanguinity in the RYK *tehsil* was also iterated when the data were analyzed through logistic regression (OR 1.34; *p* = 0.006). This study further witnessed a higher estimate of CU in subjects belonging to rural areas compared to the urban communities (OR 1.29; *p* = 0.005). This finding is in agreement with few of the previous studies including the 1990 to 1991 Pakistan Demographic and Health Survey (PDHS) [14].

In previous studies in Pakistan, high consanguinity was observed among the illiterate subjects compared with the literates [15, 16]. Our data also gave evidence that CU were higher among the illiterate subjects (OR 2.33; *p* < 0.0001). Surprisingly, however, this study also showed that the rate of CU in subjects receiving only the religious education was similar to the illiterate sample (OR 2.12; *p* < 0.0001). The record of census reports shows that the female literacy rate in RYK District is about 30 % which is the lowest in Punjab province [13, 14]. Concordant with the census data, in the present sample, there were 31.5 % women who were literate. An additional 28.9 % subjects had received only religious education. In Pakistan generally and in rural areas particularly, the girls acquire only religious education in *Madarsa* (mosque) or by a neighborhood tutor which offer very little or no formal teaching. For instance, studies have highlighted multiple causes of low literacy in Pakistan, i.e., social taboos, cultural inhibitions, societal behavior, tribal mindset, abject poverty, cultural divides, illiteracy of the parents/families, and institutional weaknesses. Furthermore, overpopulation, scarce resources and facilities, socio-economic factors, and very slowly changing attitudes, are also adding to the gravity of the situation [17]. Hence, low literacy may influence the rate of consanguinity through a number of interacting factors.

Consanguinity has been shown to be associated with the economic status of individuals [7]. In the present study, the economic status of subjects was not accessed at first hand. However, occupational status of husbands could be an indirect indicator of economic status of the subjects. With respect to the husband’s occupational category, consanguinity was observed to be highest in individuals engaged in unskilled manual, skilled manual jobs, and agriculture/farming. The individuals with such professions are usually low paid, have illiteracy or low educational levels, and belong to rural areas. On the other hand, the rate of consanguinity was the lowest in individuals working in ‘offices/services’ or engaged in ‘businesses/sales’. These categories generally comprised the subjects who were literate and economically better off than the former categories. However, in the multivariate regression model, profession of the husband was not a significant predictor of CU.

More than 98 % of all marriages recorded in the current study were ‘traditionally arranged’, in which the marriage decisions are primarily made by the couple’s parents/guardians who feel the obligation to facilitate marital contracts for their children. Reciprocal marriages are two exchanged marital unions which are also arranged by parents, and are preferentially between close relatives. In a traditional rural setup, reciprocal marriages have several potential advantages like the maintenance of family structure, wealth and property, financial benefits related to the dowry, and the ease of marital arrangements [5, 6]. Such unions may also allow wide differences between the ages of spouses. There were 34 (1.6 %) subjects who had self-arranged or arranged-love marriages; such marriages are also convened by the parents/guardians and are usually within close relatives; in these marriages bride/bridegroom or the couple has influenced the decision of parents or have engineered the situation almost entirely themselves. Such marriages had been relatively infrequent but are increasing with time [18]. Consanguinity and IC-F were observed to be highest in subjects having reciprocal (IC-F = 0.0466) and self-arranged marriages (0.0455).

In many of the rural areas of Pakistan, the common household type is extended family where CU are expected to be customary. The current study, however, showed that high consanguinity was associated with nuclear family structure. Nuclear family was also the most prominent household type in our sample. It is quite likely that in RYK, the married couple is expected to start a new household and the parents tend to stay separate. On the other hand, in upper Punjab of Pakistan, due to economic and traditional reasons, the married couple continues to stay with the parents (of husband) in the same dwelling and it leads to extended family. The extended family structure is declining primarily due to economic transition and urbanization [4]. Multivariate analyses revealed that household type was not significantly associated with CU.

One of the most significant factors observed to be associated with subjects’ consanguinity was the ‘parental marriage type’ (and this factor remained significant in all levels of statistical analyses). The IC-F in the parental generation was observed to be 0.0351 compared to the subjects 0.0355. The distribution of parental marriage types with respect to the subjects’ marriage witnessed that the parental marriage types were the predictor of subjects’ marital unions; i.e., CU in parental generation lead to a significantly higher ratio of CU among the subjects, and NCU in the parental generation lead to a significantly higher ratio of NCU among the subjects. This phenomenon depicted a traditional and cultural influence of previous marriage type on the commencement of subject marriage.

A number of studies have shown a positive association between consanguinity and live births. The results of a meta-analysis of 30 studies conducted in Asian and African countries demonstrated a higher mean number of children born in all categories of CU when compared with non-consanguineous couples [1]. The current study also reiterates this phenomenon and finds a significantly higher number of live births in the CU sample. Besides small sample size for fertility estimations, there are several explanations for this trend; for example, owing to the early age marriages in CU, the first birth occurs at an earlier age and the reproductive and fertile period remains significantly longer [1]. Likewise, a number of direct and indirect fertility determinants are potentially confounding with consanguinity, and these include but not limited to low socio-economics, religious convictions, lower contraceptive use, duration of marriage, and rural residence [19, 20].

Interestingly, the differences between the subjects having CU and the subjects with NCU were pronounced for male live births, but not for female live births. Ansari and Sinha [21] observed that consanguinity affected sex ratio, i.e., as inbreeding coefficient increases the sex ratio decreases. On the other hand, Rao [22] did not observe any significant effect of parental consanguinity on sex ratio. Studies on 1.67 million births in the USA and 0.82 million births in Denmark showed that paternal age (but not the maternal age) was associated with secondary sex ratio and significantly more male babies were born per 1000 female babies to younger fathers than to older fathers [23, 24]. Studies have suggested that social factors such as early marriage and quickly fertile couples may play a role in raising birth sex ratios in certain societies [25]. It is worthwhile to mention that Trivers and Willard [26] proposed a theory which holds that female mammals are able to adjust sex ratio in offspring, i.e., natural selection of parental ability to vary the sex ratio of their offspring. This is an interesting question to follow for prospective research in RYK and neighboring populations. Nonetheless, no difference was observed in the present study in the distribution of congenital anomalies in the offspring born to consanguineous and non-consanguineous couples. These findings are contrary to, for instance, Zlotogora [27] and many others, but are in agreement with Al-Awadi et al. [28] and El-Mouzan et al. [29]. In conclusion, these findings need to be scrutinized more critically and independently in other populations of adjoining regions.

Further, it is worthwhile to mention that for the assessment of prenatal losses, we relied on the self-reported information could be an underestimate of the actual figures. Self-reported data which is mainly based upon recall information, and may be biased towards the women’s perception of the seriousness of the health problems which in turn is influenced by the subject’s education, economic status, and accessibility to health facilities. Finally, the impact of consanguinity remains to be explored on the adult morbidity/mortality in the RYK population. A recent study in Kashmir, Pakistan has shown that consanguinity was not associated with morbidity in the adult women [30].

This study has several limitations like small sample size for fertility estimations and self-reported data on pre-natal/post-natal mortalities and hereditary malformations. This study, however, is the first report on consanguinity in one of the remote populations of Southern Punjab, Pakistan, and reveals several interesting aspects of consanguinity prevalent in this population. The presented data would be helpful in getting an insight into the socio-biological structure and health differentials of the RYK population.

## Conclusions

In conclusion, this study showed that CU were highly prevalent in Rahim Yar Khan, which is a remote district in Southern Punjab, Pakistan. The rate of consanguinity was significantly associated with certain socio-demographic variables like *Saraiki* language, illiteracy, reciprocal marriages, and parental consanguinity. The subjects with CU had a significantly higher rate of fertility, mean live births, and higher number of sons per women, as compared to the non-consanguineous group. As a prospective study, it would be worthwhile to observe the nature of congenital and hereditary malformations in younger population of RYK, and morbidities in the adult strata and their association with consanguinity.
